# Paediatric palliative care for the generalist

**DOI:** 10.4102/safp.v65i1.5722

**Published:** 2023-04-20

**Authors:** Julia F. Ambler, Christoffel H. Bell

**Affiliations:** 1 Umduduzi - Hospice Care for Children, Durban, South Africa; 2 Department of Paediatrics, Faculty of Health Sciences, University of KwaZulu-Natal, Durban, South Africa; 3 Department of Family Medicine, Faculty of Health Sciences, University of KwaZulu-Natal, Durban, South Africa; 4 Butterfly Palliative Home, Ingwavuma, South Africa; 5 Department of Health, Faculty of Family Medicine, Mosvold Hospital, University of KwaZulu-Natal, Ingwavuma, South Africa

**Keywords:** paediatric, paediatric palliative care, holistic care, health-related suffering, physical, psychological, social, spiritual, disability, life-limiting illness

## Abstract

Palliative care has been defined as ‘the active holistic care of individuals across all ages with serious health-related suffering due to severe illness, and especially of those near the end of life’. Unfortunately, palliative care and especially paediatric palliative care remain a neglected area of medicine and are widely misunderstood, with few healthcare providers having any formal training in South Africa. To relieve health-related suffering, healthcare providers must understand that the field is not limited to end-of-life care for the terminally ill, and holistic care (physical, emotional, social and spiritual) should commence at the time of diagnosis of a serious illness. It is imperative that all healthcare providers develop the knowledge and skills to provide this essential care across all levels of care and disciplines. The article aims to raise awareness and show how to practically implement palliative care through case studies.

## Background

Palliative care and especially paediatric palliative care remain a neglected area of medicine with few healthcare providers having any formal training in South Africa.^1^ According to a collaborative study by the United Nations International Children’s Emergency Fund (UNICEF) and the International Children’s Palliative Care Network, less than 5% of children with palliative care needs in South Africa, receive it.^2^ While palliative care can be provided in any setting,^3^ it is most likely that children will be down referred to facilities nearer home when curative interventions are no longer appropriate. The Hospice Palliative Care Association of South Africa suggests that a majority (84%) of hospice care is required at home, community or primary level.^4^ It is therefore imperative that general practitioners become an integral part of effective palliative care by improving knowledge and skills required to provide this care in the primary setting. The article aims to raise awareness and show how to practically implement palliative care through case studies.

## Defining paediatric palliative care

According to Lück, the World Health Organization defines palliative care appropriate for children and their families as follows:

- Palliative care for children is the active total care of the child’s body, mind and spirit, and also involves giving support to the family.
- Palliative care begins when illness is diagnosed, and continues regardless of whether or not a child receives treatment directed at the disease.
- Health providers must evaluate and alleviate a child’s physical, psychological, and social distress.
- Effective palliative care requires a broad multidisciplinary approach that includes the family and uses available community resources; it can be successfully implemented even if resources are limited.
- Palliative care can be provided in tertiary care facilities, in community health centres and even in children’s [*own*] homes.^3^

The International Association for Hospice and Palliative Care^5^ in a recent consensus statement defines palliative care further as ‘the active holistic care of individuals across all ages with serious health-related suffering due to severe illness, and especially of those near the end of life’. An important point to note is that the above definitions encompass a wide range of illnesses with potential for years of health-related suffering which requires palliative care. A common misperception among the public and healthcare providers (HCPs) is that palliative care only relates to end-of-life care and is typically for patients with advanced cancer. However, the life of the child and family is profoundly affected in all domains, physical, psychosocial and spiritual, from the time of diagnosis of a serious illness and this is when palliative care should be initiated.

In 2014, South Africa co-sponsored and participated in writing the World Health Assembly resolution 67.19, ‘Strengthening of palliative care as a component of comprehensive care throughout the life course’.^6^ This led to the launch of the National Policy Framework and Strategy for Palliative Care in 2017 which states:

Palliative care should be available to all patients as needed from birth until death and should be accessible at all levels of the health care service. Palliative care cuts across all health programs in the delivery of services.^7^

Thus, all HCPs in South Africa have a moral, ethical and legal directive to provide palliative care.

## Which children require palliative care?

Four categories of children that require palliative care have been proposed and are based on grouping conditions with similar disease trajectories. This assists HCPs to recognise when palliative care may be required and prompts thinking around where the particular child may be in the course of their illness and what the goals of care should be.

## Practical application

While the concepts of holistic and palliative care may sound reasonable, it is more difficult to put these into practice. Using case studies, we share a palliative care approach in which all domains that might require attention are assessed impeccably. These domains are physical, social, psychological, cultural and spiritual.

### Case one: Zimi

Zimi was born at 35 weeks’ gestation at a rural hospital. Her mother Sindi is unemployed and living with her mother and extended family. Zimi was found to have massive macrocephaly and transferred to her regional hospital and discussed with neurosurgery. Computed tomography scan of the brain revealed hydrocephalus with aqueductal stenosis and very thin cortical mantle. After a few weeks’ wait for a bed, she had a ventriculoperitoneal shunt inserted at the tertiary institution. She was down referred to her base hospital for ongoing care. Her discharge summary states that the baby has had a palliative shunt which means if it blocks, they will not revise the shunt because of the overall poor prognosis.

Some of the possible palliative care issues are described.

### Ethical

While ethical decision making in paediatrics is always challenging, this is particularly difficult in severe hydrocephalus.^8^ While the overall prognosis is poor with significant disability, not offering a palliative shunt will result in extremely large head size and poor quality of life. However, repeated hospital admissions, painful procedures and surgeries are burdensome for the child and family. The cost to the healthcare system is very high and may be difficult to justify balancing the prognosis, principle of distributive justice and acting in the best interests of the child. This is the reason that a decision was made to offer only one ventriculoperitoneal shunt. The earlier infants can be referred for surgical intervention, the better the prognosis and quality of life.

### Physical

**FIGURE 1:**
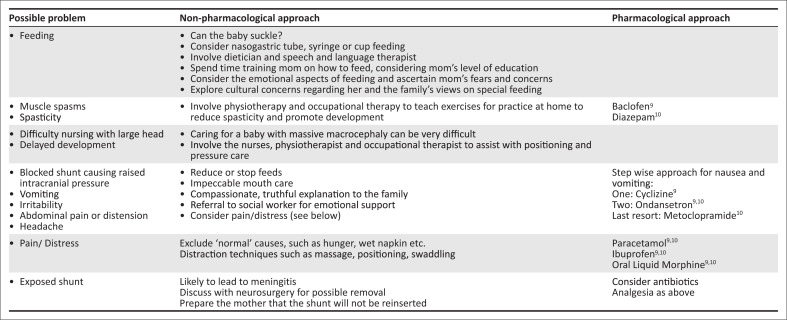
Physical needs approach.

A child such as Zimi, may have numerous physical symptoms or needs that need to be assessed and addressed. Figure 1 outlines each of these with an approach.

### Social

In this case, we have very little social history which is not uncommon in clinical practice. It is important to identify her emotional and financial support structures and to specifically enquire about involvement of the father of the baby. A father who chooses to disengage can be an enormous source of pain for a mother. Conversely, a father who wants to be involved should be included in medical discussions early in the course. It is also important to find out about extended family needs and siblings who will be directly impacted by having a sister with a disability.

Having a child with a significant health issue and disability brings numerous hospital visits, transport issues and financial strain. These parents can find themselves very isolated, having to care for the child with no respite. Referral to social worker for assistance with care dependency grant application and possible support groups can make an enormous difference.

### Psychological

As Zimi is a baby, Sindi’s psychological needs will be more prominent. It helps if the HCPs are aware of possible feelings regarding the following:

- The potential stigma, shame and non-acceptance within the community.
- Guilt that this might be her own fault.
- Grief and loss of the baby Sindi thought she was going to have.
- Coming to terms with having a child with a disability.
- Stress of having to learn new techniques on how to care for a child with special needs.

Making no assumptions, the HCP should approach the situation with curiosity to explore the emotions. Referral to social work or psychologist should be offered.

### Spiritual and cultural

An area of medicine frequently neglected is the spiritual and cultural needs of patients and their families. Health, well-being, suffering and illness are completely interwoven for many in a culturally complex society such as South Africa. To provide truly holistic care, the HCP must consider spiritual and cultural needs.

The task of offering spiritual care is that of co-creating a safe and secure or ‘sacred’ space, where the child and family can express their inner feelings or suffering and know that it is all right to do so, that they will be heard and taken seriously.^11^

Being aware that Sindi may have spiritual or cultural pain may aid in her ability to cope. She may express a sense of ‘why me?’ or ‘why my child?’; she may be angry with God or devastated that her ancestors have allowed this to happen because of some unresolved family issue. While these are not problems that can be solved medically, simply allowing the expression of the anguish to an empathetic listener can do much to alleviate some pain. An assessment tool such as FICA^12^ can be used to guide the HCP.

#### Further illustrative cases

**FIGURE 2:**
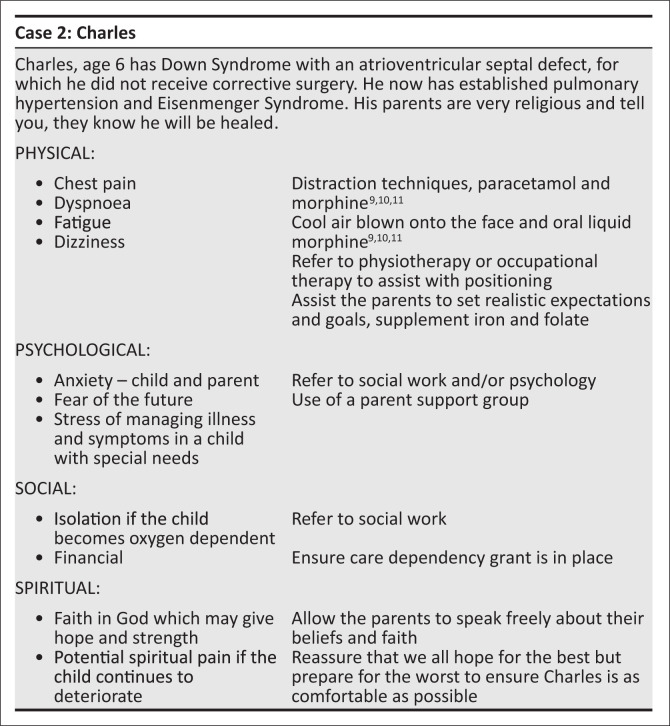
A case of trisomy 21 with Eisenmenger syndrome.

**FIGURE 3:**
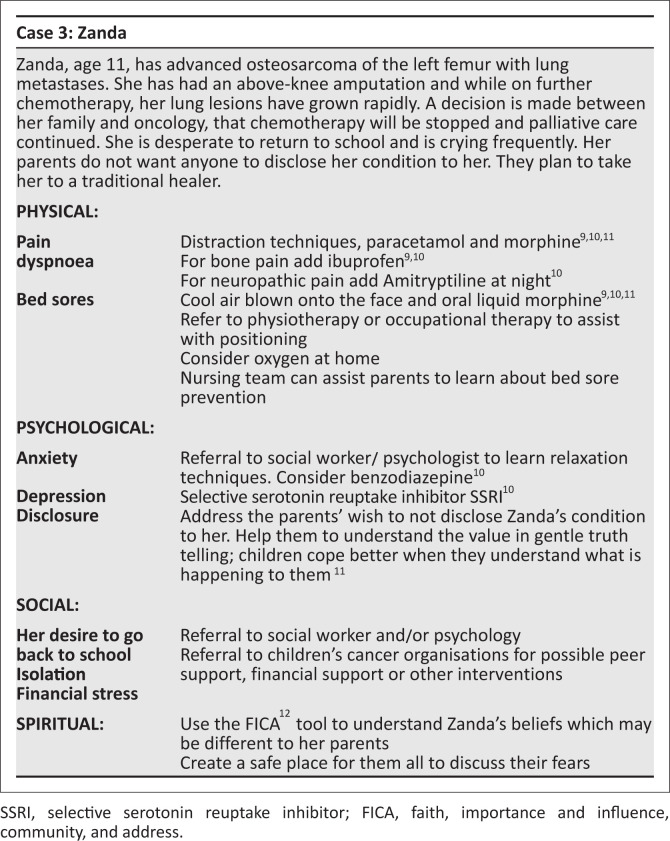
A case of advanced osteosarcoma.

Figures 2 and 3 are both summaries of paediatric palliative care cases, highlighting concerns in each domain and the suggested approach.

### Future planning

It is clear that while Zimi is not a dying child, at present she falls into category four and may have very complex care needs. There are many potential causes for suffering and ways to relieve them. With a functioning shunt, she may do relatively well, but part of palliative care is hoping for the best while we prepare for the worst. This child and family need to be supported on their journey, by recurrently assessing the needs in all domains and responding with appropriate interventions and referrals. Should the shunt block, a decision has already been made that the baby will not have a new shunt inserted and therefore the clinical condition is likely to worsen. At this time, the goal of care will need to be discussed with Sindi with less emphasis on rehabilitation and more on family support and comfort care. The family will also need to be referred for age-appropriate bereavement care for parents and siblings. This may be possible through the hospital social worker, private grief counsellors or hospice run by a non-profit organisation if available.

## Recommendations

While the absolute numbers of children with terminal illnesses are low, given the broad range of conditions requiring palliative care as in Table 1,^13^ it is likely that primary care practitioners will encounter this need. Many palliative care needs can be met through the following:

- Assessing a child with a life-threatening or life-limiting illness in all domains.
- Working collaboratively with the multidisciplinary team wherever available.
- Including the caregivers as part of the team.
- Adopting an attitude of empathetic curiosity.
- Upskilling oneself through local resources such as The Patch Academy.^14^
- Being willing to contact a palliative care specialist for advice.^14,15^

**TABLE 1:** Together for Short Lives categories of children requiring palliative care.

Category	Definition	Examples
One	Life-threatening conditions for which curative treatment may be feasible but can fail. Where access to palliative care services may be necessary when treatment fails or during acute crisis, irrespective of the duration of that threat to life.	Cancer Infections Irreversible organ failures of heart, liver, kidney where transplant is available
Two	Conditions where premature death is inevitable, but because of available treatments there may be long periods of wellness and a prolonging of life.	Cystic fibrosis Duchenne muscular dystrophy HIV on ARVs
Three	Progressive conditions without curative treatment options. Treatment is exclusively palliative and may commonly extend over many years.	Advanced metastatic cancers Mucopolysaccharidoses Trisomy 13, 18 Inoperable complex cardiac lesions
Four	Irreversible but non-progressive conditions causing severe disability, leading to susceptibility to health complications. Children can have complex healthcare needs, a high risk of an unpredictable life-threatening event or episode, health complications and an increased likelihood of premature death.	Severe cerebral palsy Multiple disabilities, such as following brain or spinal cord injury Down Syndrome Severe hydrocephalus

*Source*: Key information about children who may need palliative care, Together for Short Lives [homepage on the Internet]. [cited 2023 Jan 25]. Available from: https://www.togetherforshortlives.org.uk.

HIV, Human immunodeficiency virus; ARV, antiretroviral.

## Conclusion

Palliative care is an unavoidable part of healthcare at a primary level and deserves far more attention to ensure that a child and family are adequately cared for during an extremely vulnerable time. While the idea might seem daunting, with even basic resources, a general physician can improve the quality of the life of these children and their families. By using a holistic approach and engaging actively with the patient as well as the family members, many of the common problems in palliative care can be addressed. Often, this is determined more by the HCPs’ attitude and willingness to engage, rather than an extensive knowledge of all the intricacies of palliative care. In this field, the following saying especially rings true – a little goes a long way.
